# Impact of Atmospheric Conditions on Epistaxis Incidence

**DOI:** 10.7759/cureus.48390

**Published:** 2023-11-06

**Authors:** Alkmini Gatsounia, Georgios Schinas, Gerasimos Danielides, Michail Athanasopoulos, Spyridon Lygeros

**Affiliations:** 1 Otolaryngology–Head and Neck Surgery, University General Hospital of Patras, Patras, GRC; 2 School of Medicine, University of Patras, Patras, GRC; 3 Otolaryngology–Head and Neck Surgery, General University Hospital of Patras, PATRA, GRC; 4 Otolaryngology–Head and Neck Surgery, General University Hospital of Patras, Patra, GRC; 5 Otolaryngology–Head and Neck Surgery, General University Hospital of Patras, University of Patras, Patras, GRC

**Keywords:** temperature, humidity, meteorological concepts, climate, epistaxis

## Abstract

Purpose: A possible correlation between epistaxis occurrence and atmospheric parameters’ variation has long been hypothesized. This study aims to determine whether cumulative epistaxis incidence is related to seasonal variation and its relationship to monthly, weekly, and daily recordings of atmospheric measurements in the city of Patras, Greece.

Methods: In this retrospective study, data concerning the patients who presented with active epistaxis at the ED of a tertiary university hospital in Western Greece between January 2020 and December 2021 were collected. Only cases of spontaneous epistaxis were included in the study; patients bleeding secondarily due to a known mechanical cause, i.e., a tumor, trauma, or surgery, were excluded. The measurements of atmospheric parameters were supplied by the Department of Physics, University of Patras.

Results: In total, 230 cases of spontaneous, active epistaxis were evaluated in the ED over the course of the study. The median frequency of epistaxis presentations was two cases per week. Most of the patients were male, comprising 62.6% of the cohort, and the median age stood at 70 years, with an interquartile range (IQR) of 54 to 81 years. A minor yet statistically significant negative correlation between the incidence of epistaxis and mean relative humidity was observed both on a daily and weekly basis. Mean relative humidity emerged as a significant predictor for the incidence of epistaxis, both daily and weekly. Significantly lower mean relative humidity values were recorded during weeks with a high incidence of epistaxis cases (57.72% vs. 63.39%, p = 0.02). No discernible seasonality was observed in the frequency of epistaxis presentations to the ED.

Conclusion: A modest yet statistically significant trend toward fewer epistaxis cases was observed in conditions of higher ambient humidity during the study period in the region of Western Greece.

## Introduction

Epistaxis is a common ENT emergency that brings patients to the ED and requires immediate management. It can be either idiopathic if no apparent reason is found or secondary to trauma, foreign body implication, chronic or acute inflammation, or sinonasal tumor growth. Around 80% of all epistaxis is classified as idiopathic, with patients commonly reporting at least one underlying predisposing factor for bleeding, such as hypertension history, use of medications that impair clotting, such as anticoagulants or antiplatelet agents, and sometimes hematologic disorders, including malignancies and coagulation factor deficiencies [1]. Other systemic causes of bleeding include the use of alcohol, liver failure, renal disease, connective tissue disease, and hereditary hemorrhagic telangiectasia [2]. Epistaxis affects about 10% to 12% of the general population at least once in their lifetime, and therefore, it constitutes a significant public health issue [3]. It affects all ages with a bimodal distribution at 15 to 25 and 45 to 65 years of age, and while most cases are self-limited, some will require medical intervention and even hospitalization [4].

The majority of epistaxis cases concern the anterior region of the nasal septum and commonly originate from Kiesselbach's plexus, a highly vascularized part of the mucosa with various anastomoses between the terminal branches of the internal and external carotid arteries that is fairly susceptible to environmental inputs [5]. The initial treatment options include direct nasal compression, the application of vasoconstrictive agents, anterior nasal packing, and chemical or electrical cauterization [1]. Anterior nasal packing remains the most common treatment option for epistaxis management, as it is a fairly simple yet effective procedure [3]. Posterior nasal hemorrhage, on the other hand, is less common and typically occurs due to bleeding from the sphenopalatine artery. Usually, management with posterior nasal packing is attempted in the ED, but in severe cases or when active bleeding cannot be controlled with conservative measures, surgical intervention by ENT specialists or intravascular arterial embolization may be necessary; this suggests the life-threatening potential of epistaxis [5].

A seasonal variation in the incidence of epistaxis cases has been documented in different parts of the world. The most studied meteorological factors that seem to affect the incidence of epistaxis are temperature, atmospheric pressure, and humidity [6-10]. In Greece, only one study of this kind has been published, displaying the correlation of atmospheric factor variations to epistaxis incidence in the city of Ioannina in northwest Greece, characterized by a transient climate with Mediterranean and continental components [11]. Atmospheric measurements and their fluctuation have been linked to the incidence of disease, especially concerning cardiovascular incidents [12]. Hard outcomes, such as overall mortality, have also been explored regarding their relation to atmospheric factors [13,14].

This retrospective study aims to examine the influence of seasonality, temperature, atmospheric pressure, and humidity on the emergence of epistaxis, gathering data from the ENT department of our tertiary university hospital during the years 2020 and 2021. According to the Hellenic National Meteorological Service, the climate of Patras is characterized as Mediterranean, with mild, humid winters and warm, dry summers. Data are scarce about the relationship of this type of climate with epistaxis.

## Materials and methods

Data for this study were gathered from the Emergency Department records, focusing on all patients who visited the University General Hospital of Patras due to an active episode of epistaxis between January 1st, 2020, and December 31st, 2021. In this study, epistaxis was defined as acute, active bleeding from the nose at the time of consultation. The cumulative incidence of epistaxis for every day, week, and month of each year was thereafter calculated. Patient demographics, i.e., sex and age, were recorded, as well as the use of antiplatelets or anticoagulants for any reason. Patients whose bleeding was attributed to a previously known or established cause, i.e., hematological diseases, recent surgery, trauma, or malignancies in the nasal cavity and paranasal sinuses, were excluded. The study was conducted per the Declaration of Helsinki and Good Clinical Practice principles, and permission for the study was obtained from the institution's ethics committee (approval no. 27797).

The atmospheric measurements were recorded by the meteorological station located at our institution and were kindly provided by the Department of Physics, University of Patras. The data consisted of hourly values of the following four atmospheric parameters, measured in their respective units: temperature (oC), relative humidity (%), precipitation (mm), and atmospheric pressure (hPa).

The mean values of the above measurements for each day and week of the year were calculated using SPSS Statistics version 26 (IBM Corp., Armonk, NY, USA), and all statistical analysis described below was carried out using the same software. Descriptive statistics are displayed as such; categorical variables are described as proportions and absolute values, whereas continuous variables are described as medians and interquartile ranges (IQR) if non-normally distributed or as mean values if normally distributed. Pearson's coefficient and Spearman's rank test were used to analyze the relationship between continuous variables. Linear regression analyses were conducted to assess the association between the incidence of epistaxis on a daily and weekly basis and the mean values of the atmospheric parameters mentioned above for the respective period. To perform clinically based comparisons, weeks with a high incidence of epistaxis-related incidents were identified based on the observation of a doubling in median epistaxis incidence. Independent sample T-tests and appropriate non-parametric testing were used to compare the atmospheric values of low- and high-incidence weeks. Seasonality was examined through visual inspection of constructed scatter plots and pie charts and appropriate multiple comparison testing of quarterly cumulative epistaxis incidence. A p-value <0.05 was considered statistically significant for all analyses.

## Results

A total of 230 patients were included in the study, and their demographics, along with daily and weekly atmospheric parameters, are summarized in Table 1. The median frequency of epistaxis presentations was two cases per week. Ninety (39%) of them were treated with an anticoagulant or antiplatelet agent.

**Table 1:** Patient characteristics and mean daily and weekly atmospheric parameters during the study period (2020 to 2021)

Patients demographics	N = 230
Age (median, IQR)	70 (54-81)
Male sex (%)	144 (62.6)
Anticoagulant/antiplatelet use (%)	90 (39)
Epistaxis per day (median, IQR)	0 (0-1)
Epistaxis per week (median, IQR)	2 (1-3)
Daily atmospheric parameters (median, IQR)	
Mean temperature (^o^C)	17.18 (12.88-23.66)
Mean Relative Humidity (%)	62.83 (51.24-71.78)
Mean Precipitation (mm)	0 (0-0.01)
Mean Atmospheric Pressure (hPa)	1.009 (1.005-1.013)
Weekly atmospheric parameters (median, IQR)	
Mean Temperature (^o^C)	17.22 (12.77-24.08)
Mean Relative Humidity (%)	62.73 (55.28-69.02)
Mean Precipitation (mm)	0.01 (0-0.09)
Mean Atmospheric Pressure (hPa)	1009.2 (1006.73-1012.53)

**Figure 1:**
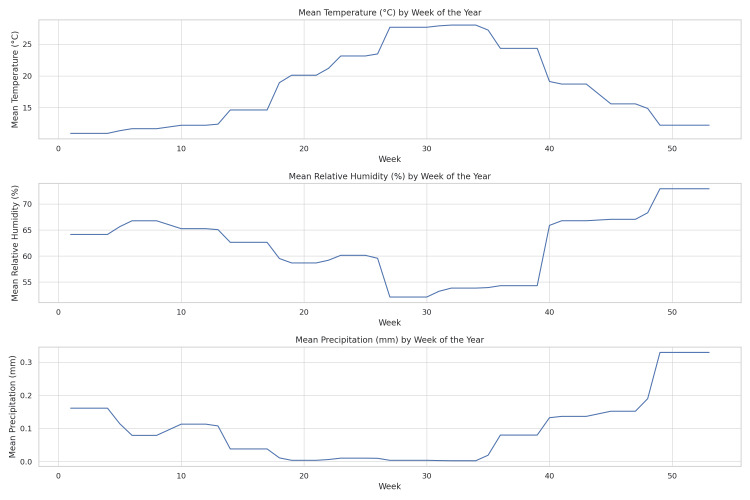
Seasonal variations in atmospheric parameters in Patras The line graphs represent the mean values of temperature in °C, relative humidity in %, and precipitation in mm averaged across all available years and plotted against the week of the year.

The comprehensive analysis of atmospheric parameters, including mean temperature, relative humidity, and precipitation, delineates distinct seasonal variations in the city of Patras. The data reveal elevated temperatures and reduced humidity during the summer weeks, coupled with milder temperatures and increased humidity in the winter, affirming existing climatological evidence for the region (Figure 1).

**Figure 2:**
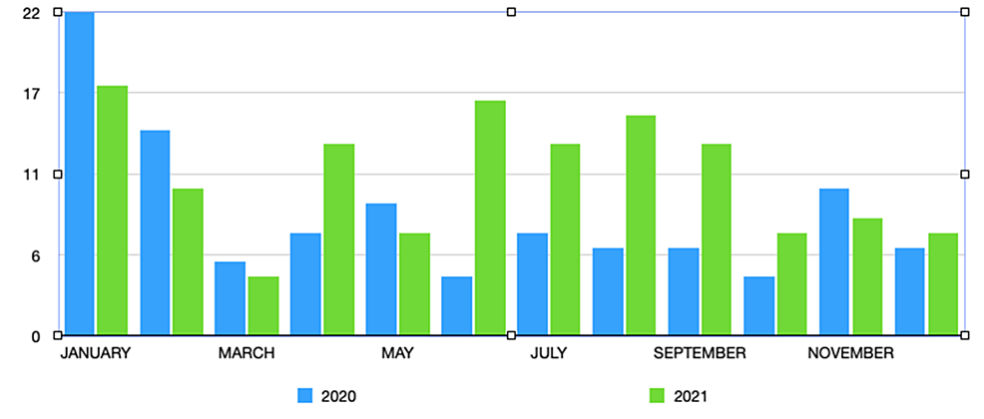
Monthly incidence of epistaxis from 2020 to 2021

No correlation was discovered between mean temperature, mean precipitation, mean pressure, and epistaxis incidence on a daily or weekly basis. No seasonal (quarterly) variation was observed in epistaxis incidence during this period of 24 months (p = 0.1) (Figure 2).

Mean relative humidity levels correlated weakly with the number of epistaxis episodes presented to the ED on both a daily (r = -0.21, p = 0.025) and weekly basis (r = -0.1, p = 0.019). Linear regression analysis, adjusted for mean temperature and precipitation, revealed that the mean relative humidity value is a significant predictor of the number of epistaxis episodes that present on a daily (β = -0.009, p = 0.048) and weekly (β = -0.58, p = 0.038) basis. The negative β-coefficients indicate that as the mean relative humidity value increases, the incidence of epistaxis episodes tends to decrease. Specifically, for each unit increase in mean relative humidity, the number of daily epistaxis episodes decreased by an estimated 0.009 (95% CI, -0.016 to -0.002). Similarly, the number of weekly epistaxis episodes decreased by an estimated 0.58 (95% CI, -0.102 to -0.13). In both models, mean temperature and mean precipitation were not significant predictors.

Moreover, we noted that the mean weekly relative humidity was significantly lower (mean relative humidity 57.72 vs. 63.39, p = 0.02) in weeks with a high incidence of epistaxis (four or more episodes presented to the ED) compared to weeks with a lower incidence of epistaxis (maximum of three episodes). The mean values of other atmospheric variables did not differ between pre-defined weeks (Table 2).

**Table 2:** Comparison between mean weekly atmospheric variables in weeks with high (four or more episodes of epistaxis) and low (maximum of three episodes) incidence of epistaxis The median number of epistaxis encountered in our ED during the two-year study period was two episodes per week, so we defined an incidence of four or more episodes per week as high.

Weekly comparison by incidence	High incidence (≥4 epistaxis episodes/week)	Low incidence (<4 epistaxis episodes/week)	p-values
Mean Temperature (^o^C) (median)	17.26	17.22	0.98
Mean Relative Humidity (%) (median)	57.72	63.39	0.02
Mean Precipitation (mm)(median)	0.07	0.01	0.73
Mean Atmospheric Pressure (hPa) (median)	1009.73	1008.81	0.22

## Discussion

Epistaxis is probably the most common ENT emergency. It is a medical event that, in most cases, does not pertain to underlying pathology or organic dysfunction; rather, it can be described as a random incident that may occur during one’s lifetime. Reportedly, up to 60% of the population will experience at least one episode of epistaxis during their lifetime [9]. Nonetheless, it is a major event that must be noted in a patient’s history and should possibly be investigated as a potential disease manifestation. In order to account for this particularity, we excluded all trauma, oncological, and surgically related incidents of epistaxis and chose to focus on the idiopathic, so-called 'spontaneous' incidents. Epistaxis tends to be more severe in the elderly population, who suffer from comorbidities such as hypertension and other cardiovascular diseases and use antithrombotic agents, e.g., antiplatelet agents and/or anticoagulants [15]. The proportion of the study population that received such medications was around 40%.

Relative humidity

Our study revealed that mean relative humidity correlates negatively with both the daily and weekly incidence of epistaxis. This suggests that more episodes of epistaxis take place on days and weeks with a lower mean relative humidity. Our findings are in line with existing literature on the link between relative humidity and epistaxis, as most studies on the matter point to an inverse relationship between the two variables [6-8,10,16]. We identified only one similar study that failed to find a significant correlation between relative humidity and the frequency of epistaxis [17].

McMullin et al. retrospectively reviewed the variation of epistaxis in a maritime city in Canada and found similar results to ours, with a negative correlation between mean daily humidity and epistaxis [8]. Two retrospective studies conducted in our neighboring country, Turkey, also suggested a negative correlation with humidity, both in children and adult populations [6,7]. Danielides et al. reported a seasonal-dependent correlation between water vapor pressure, a humidity index, and epistaxis incidence [11]. They attributed this disparity to the transient mix of Mediterranean and continental climate conditions in the region of Epirus, where the study was carried out.

The observed relationship between humidity and epistaxis incidence across various studies, despite population and climate disparities, highlights a possible pattern. In our study, although the correlation between mean daily relative humidity and epistaxis visits was relatively weak, analyzing data over an extended time period helped confirm the role of relative humidity in the cumulative incidence of epistaxis. The considerable increase in the mean relative humidity's β coefficient levels in the weekly incidence linear model could be attributed to the larger number of cases examined. However, the improved fit of the regression model concerning weekly incidence, as demonstrated by the increase in adjusted R square values (R sq = 0.052 vs. 0.012), suggested that a broader time frame may be more suitable for evaluating relative humidity's predictive value. This indicates that relative humidity levels may have a more pronounced effect on cumulative epistaxis incidence due to timely adjustments.

Temperature

The temperature may serve as an indicator of seasonal diseases such as upper respiratory tract infections (URTIs) and community-acquired pneumonia [18-20]. It is also by far the easiest measurement to be obtained with the use of a simple ambient temperature thermometer.

Although our study did not detect statistically significant correlations between temperature and the frequency of epistaxes, there are several studies supporting the idea that colder temperatures predispose to spontaneous nosebleeds [10,16,17,21] During the respiratory cycle, the nasal mucosa is alternately exposed to cool air (during inhalation) and warm air (during exhalation). The temperature of the nasal mucosa follows the fluctuation of the temperature of the air [18]. These successive changes in the temperature of the respiratory epithelium subsequently cause vasoconstriction and vasodilation of the vessels of the nose, which may lead to acute epistaxis.

Nunez et al. supported a negative linear correlation of epistaxis hospitalization rates with ambient temperatures, with more admissions taking place during the colder months in the UK [21]. On the contrary, Bray et al., who conducted a large retrospective study of the same population over a period of five years and included 1373 patients, concluded that there is no correlation between monthly mean ambient temperature and the incidence of epistaxis [22]. The disparity between findings may be partly explained by the fact that during the winter months, there is a tendency to admit patients more often due to socioeconomic reasons; hence, the admission rate may not be as accurate of a marker as the presentation rate [22].

On the other hand, studies conducted in Turkey suggest a positive correlation between epistaxis and temperature (with higher temperatures leading to more episodes of epistaxis) both in children and adult populations [6,7]. The different characteristics and comorbidities of each local population or the local climatic differences may explain the disparity between the findings [4]. However, due to considerable variation in result presentation and interpretation, no safe conclusion can be drawn.

Precipitation or atmospheric pressure

Our study revealed no significant correlation between precipitation levels, atmospheric pressure, or epistaxis incidence. In the existing literature, we were able to identify only one study supporting a negative correlation with moderate significance between monthly total rainfall and the number of spontaneous epistaxis [10].

Seasonal variation

Controversy surrounds the recorded seasonal distribution of epistaxis incidents, with most authors reporting increased rates of presentation during the cold season, while some pinpoint the increase during the hot months. In our study, no significant seasonal variation in epistaxis was noted, supporting the evidence of similarly designed studies [17,22]. Other reports, however, suggest a seasonal variation of epistaxis incidents, with the highest number of cases presenting during the winter [8,9]. An interesting explanation that may pertain to our findings relies on the combined effect of low outside temperatures and the use of indoor heating. Relative humidity is defined as the ratio of vapor pressure to saturation pressure at a given temperature. In heated indoor spaces, relative humidity tends to be lower; as a result, moisture evaporates from the upper airway, leaving the nasal mucosa dry and frail, predisposing it to epistaxis [23]. Moreover, other parameters that affect the upper airway on a seasonal basis, like URTIs, should be taken into account [4]. Inhalation of cold air causes cooling of the nasal epithelium that can decrease the mucociliary clearance and the phagocytic activity of leukocytes, predisposing them to URTIs [18]. Perhaps inflammation of the upper airway can result in a higher rate of epistaxis; however, this hypothesis requires further research.

This study has several limitations that warrant consideration. Firstly, it is a single-center, retrospective study that is geographically constrained to Western Greece, which limits the generalizability of the findings, as varying climates and environmental conditions in other regions could yield different outcomes. Additionally, our patient population was solely comprised of individuals who presented with epistaxis at the ED of a tertiary-level hospital, which serves as a referral center for Western Greece. Therefore, cases treated in regional lower-tier hospitals, primary care centers, and private practices are not accounted for in this study. Moreover, with the COVID-19 pandemic causing a decrease in ED patient visits, the recorded incidence of epistaxis may not fully represent the usual patient traffic. Regarding patient selection, our analysis considered those on anticoagulants. It was vital due to the frequent occurrence of epistaxis in this group. While this adds a measure of real-world practicality to our findings, it might slightly complicate the interpretation of the correlation between epistaxis incidence and atmospheric parameters. Finally, while we focused on the daily, weekly, and monthly mean for each atmospheric variable in our analysis, different outcomes may emerge if alternative time frames or central tendency measurements are employed.

## Conclusions

In summary, our investigation revealed a modest inverse relationship between the cumulative incidence of epistaxis and mean relative humidity on both a daily and weekly basis. According to our statistical modeling, the proportion of variability in epistaxis incidence explained by the mean relative humidity is limited, accounting for an estimated 1% to 5% of the total observed variance. To strengthen the generalizability of these results, future studies should be conducted in diverse climatic conditions and potentially incorporate additional environmental variables or patient-specific factors.
